# Supplementary material for: Firearms and violence in Europe–A systematic review
Source: PLoS One. 2021 Apr 14;16(4):e0248955. doi: 10.1371/journal.pone.0248955 (PMC8046231; doi:10.1371/journal.pone.0248955)
Supplement: Attachment — Submitted filename: Response to Reviewers.docx [file pone.0248955.s004.docx]

Dear Dr. Topaz and reviewers,

Thank you for giving us the opportunity to revise our manuscript ‘Firearms and violence in Europe – a systematic review’. Your comments were constructive and helpful. We have adapted all of your suggestions in our revised manuscript. A more detailed response to each comment made by the reviewers can be found below and changes are highlighted in the revised manuscript.

Sincerely,

Katharina Krüsselmann

Pauline Aarten

Marieke Liem

**Reviewer and Editor Comments, Authors’ Responses and Changes**

*line numbers correspond with the clean version of the revised manuscript

Editor

Comment 1:	Please ensure that your manuscript meets PLOS ONE's style requirements,
 including those for file naming.

*Changes:	We have adapted the style of the review according to the requirements.*

Comment 2:	Please include additional information about your study quality assessment
 method. In your manuscript, you refer to ref. 13 for more information,
 however, If materials, methods, and protocols are well established, authors
 may cite articles where those protocols are described in detail, but the
 submission should include sufficient information to be understood
 independent of these references

*Response:	Thank you for this valuable comment. We have added a description of the
 quality assessment methods in the review	.*
*Changes:	Changes made in lines 154-160.*

Comment 3:	We note that you have indicated that data from this study are available upon
 request. PLOS only allows data to be available upon request if there are legal
 or ethical restrictions on sharing data publicly.

*Response:	We have made the statement that data is available upon request, as not all
 studies included in this systematic are published open access. We do, however,
 not have specific datasets from these studies.*

Reviewer 1

Comment 1:	There is a need to either restate the second goal of the paper (lines 68-71), or
 to add more information that supports it. Because the papers have few
 common findings, it does not seem reasonable to use this work to inform
 potential effects and future trends. The reasons behind this incongruence are
 explained in the discussion. If this is still a goal of this manuscript, then far
 more information needs to be added and synthesized to make specific
 statements. Conversely, if this goal can be restated, this is no longer a major
 issue.

*Response:	First of all, thank you for your clear comments throughout your review. It is
 indeed clear that we did not meet our second goal, given the low quantity of
 eligible studies and their methodological characteristics, which did not allow
 for us to make any clear conclusions regarding the effects of firearm legislation.
 We have restated our goal to match the information gained from this systematic
 review more clearly.*

*Changes:	We have changed our second goal to: ‘inform researchers, practitioners and
 policymakers in the domains of public health and criminal justice about the
 current state of knowledge regarding the association between firearm
 availability and violence, remaining lacunae of knowledge to be filled by and
 methodological challenges to be addressed by future research’.*

*The text has been adapted in the introduction (lines 79-83) and the conclusion.*

Comment 2:	Another major issue is the number of studies reviewed. A sample size of six
 makes it difficult to accept any conclusions, even if the conclusions are that
 there are not enough studies. This can be remedied by either relaxing the
 inclusion criteria and including more studies or discussing some of the results
 from excluded studies to give more support to specific conclusions.

*Response:	We agree that the low N of eligible studies presents a disadvantage, as already
 stated in the response to your first comment. In the preparation of the search
 protocol, we have tried different inclusion criteria and agreed on the necessary
 practical, as well as logical inclusion criteria, such as location and timeframe
 that allow us to answer the research question. To overcome some of the
 limitations of the low N, we placed our findings in a broader context by
 comparing them with findings from international studies that include, amongst
 others, European data in the discussion. To emphasize the relevance of those
 studies, we now added a more concrete summary of those international studies
 in the discussion, as well as a short comparison of the findings from the global
 studies with the included European studies. A challenge in such a comparison
 – yet at the same time important conclusion – is that comparisons are difficult
 due to the varying results found in each of the studies, mainly due to socio-
 economic and cultural differences between the countries under study. We have
 now explicitly stated this in the discussion. Moreover, we hope that the low
 number of empirical studies will show the need for methodologically robust
 research on this topic.*

*Changes:	We have added a paragraph (lines 315-329) in the discussion with some
 additional observations.*

Comment 3:	For the Introduction (lines 47-71), include more information regarding firearm
 use in Europe. For non-European readers, it would be helpful to understand
 more about the legislation that is in place and the culture surrounding firearms
 in Europe. Some of this is discussed in lines 258-262, but may be better suited
 as background information. Similarly, include more information about past
 analyses of legislation. Particularly, The Science of Gun Policy (Smart 2020)
 conducts these kinds of studies in the United States and there are likely others.
 Highlighting some of these past works and the methods used to synthesize
 multiple papers or pieces of legislation will give support to your claims.

*Response:	Thank you for this valuable comment.*

*Changes:	We have added a summary (lines 59-74) of relevant research on gun
 culture and -legislation in Europe to the introduction.*

Comment 4:	For Table 1, add the title of the manuscript to the table. Even though the
 bibliography contains this information, readers are more likely to look at the
 studies if the title is readily available.

*Response:	We agree.*

*Changes:	Titles of studies are added in table 1.*

Comment 5:	Clarify the differing levels of analysis and potentially define the study design
 types (Table 1, Table 2, lines 169-173). Especially when discussing levels of
 analysis, some readers may understand ``individual'' to mean specific people
 and it is unclear whether this is the case in your analysis.

*Response:	This is an important comment, thank you. To clarify the kind of data used in the
 different studies, and to avoid misunderstanding, we have adapted the
 information presented in table 2 to reflect whether a study was conducted
 nationally vs. cross-nationally, using aggregated vs. non-aggregated data.
 Moreover, we have added a column in table 2, describing the research design
 of each study.*

*Changes:	In table 2, the level of analysis has been adapted. A clarification has also been
 added in lines 170-178. A column ‘study’ design was added in table 2.*

Comment 6:	Within the conclusion and discussion, present some of the ways in which
 future studies could meet inclusion criteria for a future review. There are many
 mentions of the need for consistency across studies, but tying back in the
 reasons why certain studies were or were not included in your analysis would
 give more guidance to researchers in the field.

*Response:	In addition to the suggestions for future research mentioned in the
 original manuscript, we have added two additional suggestions, which – we
 believe – would increase the quality of future research: first, although various
 proxies of firearm availabilities are used, there is no examination of the validity
 of these proxies. Therefore, we suggest that future studies should focus on
 available proxies, such as data from the Small Arms Survey, the
 International Crime Victim Survey or European firearm suicide data. Such a
 research has been carried out in the past in the US context. Our second
 suggestion is that more studies need to carry out empirical tests to assess the
 link between firearm availability and -violence. Although various studies on
 fatal and non-fatal violence in Europe mention firearm availability as a factor,
 most assumed the association between availability and violence, rather than
 testing such a link.*

*Changes:	We have added above described suggestions for future research in lines 357-
 365 in the revised manuscript.*

Reviewer 2

Comment 1:	The authors mention that they ruled out 25 papers where they were unable to
 get a full-text copy. Have they succeeded in getting any of these in the months
 since? It seems like Feb - April was a hard time everywhere, and so the
 authors they emailed might have dropped the ball on writing back. Were those
 authors ever emailed again?
*Response:	This is a very good point. The pandemic has changed all of our private and
 professional rhythms and it would be more than understandable if our
 contacted authors were not able to respond to our initial email. Therefore, we
 have send out another email again, on November 10^th^, as well as follow-up
 reminder on November 24^th^. As a result, we have received two more studies,
 which we have assessed according to our eligibility criteria. Unfortunately, we
 had to exclude both studies on the merit that they did not (exclusively) include
 European data. We have adapted the text, as well as the PRISMA flow chart
 accordingly.
Changes:	Numbers of studies received and excluded were adapted in the text (lines 142-
 147) and the PRISMA flow chart.*

Comment 2:	In the Outcomes column of Table 2, many of the papers are summarized using
 overly causal language. For example, when describing reference [14], the table
 writes “Removing firearms from private homes of National Guards reduces
 firearm homicides” but [14] uses less causal language, saying the laws “could
 have contributed.” Similarly, in describing [15] the table says “Stricter firearm
 policies lead to less firearm and non-firearm homicides” but the methods of
 [15] (linear and Poisson regression) do not support causal conclusions.
 Similarly, “lead to” is used in the description of [16] and [18], while for the
 description of [17], “Both owning and carrying a firearm increased the risk of
 injuring another person intentionally” again suggests a causal link. In all these
 cases, the authors of those studies are careful to avoid overly causal language.
 Furthermore, all six of the study designs (except possibly the quasi-
 experimental design of [17]) do not seem capable of proving causation. I
 encourage the authors to modify their language to strip out unsupported causal
 claims. For example, ‘lead to” can be replaced (in both Table 2 and the
 subsequent discussion of the six papers) with “is associated with.” As the
 authors themselves write in the first paragraph of the introduction, “causal
 links between the prevalence of firearms and violence remain unclear.” It’s
 best not to accidentally insert causal claims where they were not supported by
 the papers in question. For example, [14] is an observational study. Most of
 the six papers use time-series methods and observe that gun violence dropped
 when new laws were put into effect. But this approach ignores the possibility
 that culture is a confounding variable that causes both the change in laws and
 the drop in gun violence.
*Response:	This is a very valuable comment, thank you. Although we have tried to adapt
 the language used by the authors of the included studies, we see that some of
 the statements made in our review require careful revision to avoid making
 causal claims that are not supported by the data. We have critically and
 carefully reviewed the language used throughout and adapted where necessary.
Changes:	Language has been adapted throughout the analysis, to avoid overly causal
 claims that are not supported. For example in table 2.*

Comment 3:	My last comment before moving on to the line-by-line remarks is that I want
 to encourage the authors to take up a similar literature review for the interplay
 between firearm availability and suicide rates, as well as accidental shootings.
 Since you’ve already done the first step, of combing through all the papers,
 this would be easier for you than for other researchers and it’s arguably just as
 important as the systematic review you’ve just completed.
*Response:	We agree that such a review would be a great addition to the body of scholarly
 work. For our study, however, we have decided to exclude accidents and
 suicides, due to our expectation that those forms of violence have different
 underlying causes. However, we will keep the extracted literature and hope to
 continue and explore the association of firearms and suicides and accidents in
 the future	.
Changes:	No changes made.*

Comment 4:	Around line 116, the authors write that their approach was inspired by [2] and
 [6]. It would be good to say a word here about why this paper is different from
 [2] and [6], and still needed despite [2] and [6] already doing a systematic
 review of literature related to firearm availability. I’m guessing it’s because
 [2] includes data from outside Europe and does not include control variables,
 and [6] includes data from outside Europe, but this would be good to say.
*Response:	A good point. We have added the reason to our text.
Changes:	See line 129-130.*

Comment 5:

- Line 162 has a dash that it doesn’t seem to need.
- Same in the Outcomes column for the Kapusta paper and for the K ̈onig paper.
- Same on lines 304 and 305.
- Line 241: “This systematic review sought to assessing all studies”
- Line 262: should “incongruent legislations” be “legislation” instead? I’m not sure.
- Line 282, should “in academic literature” be “in the academic literature”?
- Line 295, I think either the dash after ‘motorcycle’ should be dropped, or you have to add another dash after ‘gangs’ on line 296.

*Response:	Thank you for your detailed observations. The dashes were included to indicate
 (for example in line 304) that we want to make a statement about firearm
 homicides, not homicides in general. We left ‘legislations’ instead of
 ‘legislation’, given that many European countries – namely EU member states
 – follow the same framework of regulation, yet national differences exist.
Changes:	Adapted in the text.*
