# Supplementary material for: Firearms and violence in Europe–A systematic review
Source: PLoS One. 2021 Apr 14;16(4):e0248955. doi: 10.1371/journal.pone.0248955 (PMC8046231; doi:10.1371/journal.pone.0248955)
Supplement: Attachment — Submitted filename: Response to Reviewers.docx [file pone.0248955.s005.docx]

Dear Dr. Useche and reviewers,

Thank you for giving us the opportunity to revise our manuscript ‘Firearms and violence in Europe – a systematic review’. Your comments were constructive and helpful. We have adapted all of your suggestions in our revised manuscript. A more detailed response to each comment made by the reviewers can be found below and changes are highlighted in the revised manuscript.

Sincerely,

Katharina Krüsselmann

Pauline Aarten

Marieke Liem

**Reviewer and Editor Comments, Authors’ Responses and Changes**

*line numbers correspond with the clean version of the revised manuscript

Editor

Reviewer 1

Comment 1:	Line 49: Put this statistic in per capita form and potentially compare it to the
 continental United States or another similar region. As a researcher from the
 U.S., 7,000 seems like a small number, but is even smaller (relatively) when
 considering the population of continental Europe. Comparing it to another
 country will put it into perspective for readers.

*Response:	Thank you for this detailed comment. We agree that it is important to
 understand the issue of gun violence in the geographical context in which it
 takes place, one of the main points we are trying to make in this article. We have
 added your suggestions.*

*Changes:	We have added a calculation of the 7000 firearm deaths in Europe per 100.000
 population in line 49. Moreover, we added half a sentence in lines 54-55 with
 the same calculation for the United States.*

Comment 2:	Line 60: Change ``vultures'' to ``cultures.'' This is an understandable typo, but
 I wanted to be sure it was highlighted!

*Changes:	We have corrected this mistake in line 61.*

Comment 3:	Lines 157 - 160: italicize or bold good, fair, and poor when initially defining
 them. This will make it easier to find the definitions for readers as they work
 through the review

*Changes:	We have italicized ‘good’, ‘fair’, and ‘poor’ in lines 161 and 162, hoping that
 it will make the manuscript more accessible.*

Comment 4:	General: Extend the captions for figures and tables. Add a brief statement
 about what should be taken away from each table and figure so that they
 would be able to stand alone, without the text of the main manuscript

*Changes:	We have extended the captions for the tables (line 170-171, line 190-191) to
 include the specific factors examined in the tables. To stay in line with previous
 studies published by PLOS ONE, we have opted not to add a concluding
 statement of each table.*

Reviewer 2

Comment 1:	Line 61: please capitalize Constitution
*Changes:	We have corrected this mistake in line 62.*

Comment 2:	Line 62: "baring" should be "bearing"	*Changes:	This mistake is corrected in line 63.*

Comment 3:	Lines 154-157: Did you mean to have (1)-(5) display as one long sentence, or
 did you mean to have one item per line? I think it would be easier to read if it
 was one item per line, but it's up to you.
*Response:	We agree that the text can be made more accessible by separating the different
 items.
Changes:	Items were separated in lines 155-160*

Comment 4:	Line 316: perhaps this colon should be a period since the next word is
 capitalized
*Changes:	See line 321.*

Comment 5:	Line 323: there is a hyphen before a comma that does not seem to belong

*Changes:	Hyphen is deleted in line 331.*
