# Supplementary material for: Firearms and violence in Europe–A systematic review
Source: PLoS One. 2021 Apr 14;16(4):e0248955. doi: 10.1371/journal.pone.0248955 (PMC8046231; doi:10.1371/journal.pone.0248955)
Supplement: Attachment — Submitted filename: referee report on gun paper for PLOS One.pdf [file pone.0248955.s003.pdf]

Referee report on Firearms and violence in Europe - A systematic review

This paper fills an important gap in the literature, by conducting a systematic review, following the PRISMA guidelines, of the corpus of papers that use data to study the Firearms Availability Hypothesis in Europe. This hypothesis states that greater availability of firearms is associated with more incidents of firearm-enabled criminal violence. The authors exhaustively searched nine databases and some 8,000 papers to find all papers satisfying their desiderata. The result is six papers that address this question, using data, during the appropriate time frame, using control variables, and producing robust results. The authors do an excellent job explaining their methodology, describing these six papers, distilling conclusions, and making recommendations for future work. I expect that future papers will build on and cite this paper.

I have two substantive questions/comments for the authors, and then a small number of line by line comments.

(1) The authors mention that they ruled out 25 papers where they were unable to get a full-text copy. Have they succeeded in getting any of these in the months since? It seems like Feb - April was a hard time everywhere, and so the authors they emailed might have dropped the ball on writing back. Were those authors ever emailed again?

(2) In the Outcomes column of Table 2, many of the papers are summarized using overly causal language. For example, when describing reference [14], the table writes “Removing firearms from private homes of National Guards reduces firearm homicides” but [14] uses less causal language, saying the laws “could have contributed.” Similarly, in describing [15] the table says “Stricter firearm policies lead to less firearm and non-firearm homicides” but the methods of [15] (linear and Poisson regression) do not support causal conclusions. Similarly, “lead to” is used in the description of [16] and [18], while for the description of [17], “Both owning and carrying a firearm increased the risk of injuring another person intentionally” again suggests a causal link. In all these cases, the authors of those studies are careful to avoid overly causal language. Furthermore, all six of the study designs (except possibly the quasi-experimental design of [17]) do not seem capable of proving causation. I encourage the authors to modify their language to strip out unsupported causal claims. For example, ‘lead to” can be replaced (in both Table 2 and the subsequent discussion of the six papers) with “is associated with.”

As the authors themselves write in the first paragraph of the introduction, “causal links between the prevalence of firearms and violence remain unclear.” It’s best not to accidentally insert causal claims where they were not supported by the papers in question. For example, [14] is an observational study. Most of the six papers use time-series methods and observe that gun violence dropped when new laws were put into effect. But this approach ignores the possibility that culture is a confounding variable that causes both the change in laws and the drop in gun violence.

My last comment before moving on to the line-by-line remarks is that I want to encourage the authors to take up a similar literature review for the interplay between firearm availability and suicide rates, as well as accidental shootings. Since you’ve already done the first step, of combing through all the papers, this would be easier for you than for other researchers and it’s arguably just as important as the systematic review you’ve just completed.

Line-by-line editorial remarks

(1) Around line 116, the authors write that their approach was inspired by [2] and [6]. It would be good to say a word here about why this paper is different from [2] and [6], and still needed despite [2] and [6] already doing a systematic review of literature related to firearm availability. I'm guessing it's because [2] includes data from outside Europe and does not include control variables, and [6] includes data from outside Europe, but this would be good to say.
(2) Line 162 has a dash that it doesn't seem to need.
(3) Same in the Outcomes column for the Kapusta paper and for the König paper.
(4) Same on lines 304 and 305.
(5) Line 241: "This systematic review sought to assessing all studies"
(6) Line 262: should "incongruent legislations" be "legislation" instead? I'm not sure.
(7) Line 282, should "in academic literature" be "in the academic literature"?
(8) Line 295, I think either the dash after 'motorcycle' should be dropped, or you have to add another dash after 'gangs' on line 296.
